# Diversity, antibacterial activity and chemical analyses of gut-associated fungi isolated from the *Crocothemis servilia*

**DOI:** 10.3389/fmicb.2022.970990

**Published:** 2022-09-16

**Authors:** Pu Cui, Lijun Liu, Zhongdi Huang, Shuping Shi, Kun Kong, Yinglao Zhang

**Affiliations:** ^1^School of Life Sciences, Anhui Agricultural University, Hefei, China; ^2^State Key Laboratory of Tea Plant Biology and Utilization, Anhui Agricultural University, Hefei, China

**Keywords:** *Crocothemis servilia*, fungal diversity, insect-associated fungi, antibacterial activity, secondary metabolites

## Abstract

Insect-associated fungi are a potentially rich source of novel natural products with antibacterial activity. Here, we investigated the community composition and phylogenetic diversity of gut-associated fungi of the dragonfly (*Crocothemis Servilia*) using a combination of culture-dependent and culture-independent methods. A total of 42 fungal isolates were obtained from the guts of the dragonfly, which belonged to four classes and thirteen different genera. Amplicon sequencing analyses revealed that the fungal communities were more diverse, and a total of 136 genera were identified and dominated by the genera *Wojnowiciella* and *Phoma*. The antibacterial bioassay showed that five fungal crude extracts of representative isolates have shown antibacterial activities. Among them, the extract of *Phoma* sp. QTH17 showed the best antibacterial activities against *Escherichia coli*, *Micrococcus tetragenus*, and *Staphylococcus aureus* with the disc diameter of inhibition zone diameter (IZD) of 6.50, 10.80, and 8.70 mm, respectively. Chemical analysis of *Phoma* sp. QTH17 led to the discovery of five known compounds, including ergosterol **(1)**, 3-Chlorogentisyl alcohol **(2)**, epoxydon **(3)**, epoxydon 6-methylsalicylate ester **(4)** and mannitol **(5)**. Among them, the compound **3** exhibited potent antibacterial activities against *E. coli*, *M. tetragenus*, and *S. aureus* with the IZD of 7.00, 14.00, and 12.50 mm, respectively, which were slightly weaker than those of the positive gentamicin sulfate with the IZD of 11.13, 18.30, and 12.13 mm, respectively. In conclusion, our results confirmed that the diversity of gut-associated fungi of *C. Servilia* could be expected to explore the resource of new species and antibacterial substances.

## Introduction

Insects are the most abundant group of animals and coexist with various microorganisms (Park et al., 2018). Due to the complexity and diversity of their habitats and diets of their insect partners, the insects-associated microorganisms are also diverse (Long et al., 2022). Fungi are one of the largest groups of eukaryotic organisms on Earth and play a significant role in insect development and fitness (Gibson and Hunter, 2010; Sarikaya Bayram et al., 2010). The gut microbiome in insects has been a hot research topic in microbiology, and which diversity determines their host functions (Xia et al., 2020). The culture-dependent and culture-independent approaches have recently been used to evaluate the fungal composition and diversity of insects’ guts. For example, two approaches were used to assess gut fungal diversity in termites, and characterize gut fungal communities of the wild and laboratory-reared ghost moth larvae (Makonde et al., 2013; Liu et al., 2021). Although there are many studies on the diversity of fungi associated with insects, most are only focused on social insects.

Insect-associated fungi have been isolated from various insects, including termites, honeybee, leaf-cutting ants, *Dactylopius*, caterpillars, and black soldier fly (Pereira et al., 2016; Vera-Ponce de León et al., 2016; Tauber et al., 2019; Višňovská et al., 2020; Nagam et al., 2021; Tegtmeier et al., 2021). Yeast species were the most common fungi in the insect gut, and have beneficial effects on their host, such as immune system enhancement, antimicrobial activity, and nutrient supplementation (Stefanini, 2018; Meriggi et al., 2020). Some filamentous fungi were also frequently isolated, including *Trichoderma*, *Pyrenochaeta*, *Fusarium*, *Acremonium*, *Chaetomium*, and *Penicillium* (Xu et al., 2020; Nagam et al., 2021). Those insect gut-associated fungi were the resource of new species and novel bioactive products, and have attracted the wide attention of scholars (Zhang et al., 2008; Moubasher et al., 2017). For instance, two novel yeast species isolated from the guts of termites play a symbiotic role in the host concerning xylan degradation and production of vitamins (Handel et al., 2016). Two novel compounds were purified and identified from *Trichoderma harzianum* isolated from the gut of *Pantala flavescens*, one of the compounds has displayed moderate antibacterial activity against *Bacillus subtilis* (Zhang et al., 2020). Some active compounds with antibacterial activity against important human pathogens have been found from insect-associated fungi, including long-horned grasshoppers, termites, and black soldier fly (Li et al., 2014; Correa et al., 2019; Nagam et al., 2021). Thus, there is an enormous potential for the production of secondary metabolites with biological activities by insect gut-associated fungi.

As a flying invertebrate, the dragonfly belongs to the class Insecta and the order Odonata in the phylum Arthropoda. Additionally, the dragonfly is a flesh-eating insect, and beneficial for agriculture and forestry (Maoka et al., 2020). Dragonfly guts microbiome has an important impact on host ecology and evolution and is related to dietary differences (Nair and Agashe, 2016; Nobles and Jackson, 2020). Gut-associated fungi of dragonfly were highly diverse and discovered as the resource of bioactive compounds (Shao et al., 2015; Lu et al., 2017; Zhang et al., 2020). *Crocothemis servilia* is one of Asia’s most widely distributed dragonfly species, mature males and most females are scarlet-red and yellow, respectively (Joshi et al., 2020). However, no study reported the gut fungal diversity of the dragonfly. Our understanding of the species range, biological activity, and secondary metabolites of these gut-associated fungi of the dragonfly is still limited. Here, we investigated the gut fungal community of the dragonfly by using both culture-dependent and culture-independent methods. The antibacterial activity and secondary metabolites of the gut-associated fungi were also evaluated.

## Materials and methods

### Sample collection

Dragonflies (*C. servilia*), which were medium-sized and authenticated by assistant professor Xin Yu (College of Life Sciences, Chongqing Normal University), were collected from a pond in Anhui Agricultural University, Hefei, China (GPS: 31°86′N, 117°26′E) in September 2018. The collected samples were rapidly transported to the laboratory and starved for 24 h, then some samples were stored at –20°C for fungal isolation and at –80°C for DNA extraction, respectively.

### Fungal isolation

Two dragonfly individuals (*C. servilia*) were surface-sterilized in 75% ethanol for 3 min, followed by rinsing three times with sterilized water. Subsequently, the guts of samples were dissected and homogenized with 5 mL of sterilized water. Finally, the homogenates were diluted in a 10-fold series (i.e., 10^–1^, 10^–2^, 10^–3^), and an aliquot of 100 μL was spread onto three different isolation media: Potato dextrose agar (PDA) (Xu et al., 2020), malt extract agar (MEA) (Shao et al., 2015), ISP medium no. 2 (ISP 2) agar (Xu et al., 2020). All isolation media were added with ampicillin (10 mg/mL) and streptomycin (10 mg/mL) to suppress the growth of bacteria. Pure colonies of fungi from the appropriate dilution were transferred into a new PDA medium and incubated aerobically at 28°C. All these isolated fungal strains were maintained on PDA slants at 4°C until use. The fungi were also preserved on glycerol suspensions (25%, v/v) at –80°C.

### Taxonomic identification of fungal isolates

All fungal isolates were identified by molecular techniques and morphological characteristics (Li et al., 2014; Xu et al., 2020). Each fungus was cultured in PDA plate at 28°C for 3–4 days. Then the fungal genomic DNA was purified using the Fast DNA Extraction Kit [Tiangen Biotech (Beijing) Co., Ltd., Beijing, China] according to the manufacturer’s instructions. As mentioned before (Shao et al., 2015), PCR amplification for the 5.8S rDNA gene sequence was performed using the universal fungal primers ITS1 and ITS4. The quality was visualized on 1% agarose gel by electrophoresis. Qualified products amplified from PCR were sent to General Biosystems (Anhui) Co., Ltd. for sequencing. All achieved sequences’ affiliations were compared with that of available data in the NCBI database using the BLAST program. Sequence alignment and Neighbor-joining phylogenetic tree were constructed using MEGA software version 5.0. Bootstrap replication (1,000 replications) was used to assess the topology of the phylogenetic tree (Felsenstein, 1985). The ITS sequence data were submitted and deposited in GenBank under accession numbers ON248244-ON248285.

### Culture-independent community analysis

Six dragonfly individuals (*C. servilia*) were surface-sterilized in 75% ethanol for 3 min, followed by rinsing three times with sterilized water. Then, the entire guts were removed with sterile forceps, and the gut total genome DNA of the two samples was extracted using the CTAB method (Doyle, 1987). The concentration and purity of DNA were monitored on 2% agarose gels. We generated three samples for amplicon sequencing in this study. Libraries were prepared and sequenced using specific primers ITS5-1737F and ITS2-2043R to amplify region ITS1 of the ITS gene of fungi. At last, the libraries were sequenced on an Illumina NovaSeq platform to generate approximately 250 bp paired-end reads.

Paired-end reads were merged using FLASH V1.2.7 and analyzed using QIIME V1.9.1. We used the QIIME V1.9.1 and UCHIME Algorithm for quality control, filtering of chimeric sequences, and obtaining effective tags (Caporaso et al., 2010; Edgar et al., 2011; Haas et al., 2011). Sequences having greater than 97% similarity were assigned to the same operational taxonomic units (OTUs) using the software Uparse v7.0.1001 (Edgar, 2013). The taxonomy of each representative sequence was assessed by using the MUSCLE software V 3.8.31, and comparison against the Unite database based on the blast algorithm (Edgar, 2004). Alpha diversity and species annotation were based on phylogenetic relationship construction. Raw data is accessible from NCBI’s short read archive under accession number PRJNA827522.

### Antibacterial activities

The filter paper dispersion method was used to evaluate the antibacterial activity of some representative crude extracts (Xu et al., 2020). Each fungus was inoculated in a 250 mL Erlenmeyer flask containing 150 mL of ME liquid medium and cultured for 7 d at 180 rpm and 28 ± 0.5°C. The culture broth filtrate was exhaustively extracted three times with ethyl acetate (EtOAc, 1:1, v/v). Then, the ethyl acetate extract was evaporated under vacuum to yield fungal crude extract. The fungal crude extract was dissolved in acetone to get a concentration of 6 mg/mL, and 5 μL of the tested solution was pipetted onto a sterile filter paper disc (6 mm in diameter), which was placed onto pre-prepared LBA medium (1% tryptone, 1% NaCl, 0.5% yeast extract, 2% agar) plates with a suspension of the pathogenic bacteria. The pathogenic bacteria were *Escherichia coli* (ATCC8739), *Micrococcus tetragenus* (ATCC35098), and *Staphylococcus aureus* (ATCC6538). The sterile paper disc treated with acetone alone and gentamicin sulfate were served as a negative and positive control, respectively. Each inhibition assay was repeated three times and evaluated by measuring the diameters of inhibition zone diameter (IZD) (in mm).

### Secondary metabolite characterization

One fungal strain QTH17 was selected for purification and identification of compounds in this study. Strain QTH17 was grown on PDA medium plates for 3–4 days at 28°C. Then the fresh mycelia (3 plugs of 5 mm) were inoculated into 250 mL Erlenmeyer flasks containing 150 mL of ME liquid medium and cultivated for 4 days at 28°C with shaking at 180 rpm. After that, aliquots (15 mL) of the culture were transferred into 1,000 mL Erlenmeyer flasks filled with 400 mL of the same medium and cultured at 28°C for 7 days with shaking at 180 rpm. A total of 16 L of the fermentation broth of QTH17 was filtered and extracted with EtOAc three times (3 × 16 L). The EtOAc phase was subsequently concentrated *in vacuo* to obtain 3.0 g of crude extract.

The crude extract (3.0 g) was dissolved in 10 mL solvent of methanol/methylene chloride (2/8, v/v) and mixed with 6.0 g dry silica gel powder (100–200 mesh, Qingdao Haiyang Chemical Co., Ltd., Qingdao, China). Then, the mixture was passed through silica-gel column chromatography (60 g, 200–300 mesh, Qingdao Haiyang Chemical Co., Ltd., Qingdao, China; Φ: 60 mm, the height of the silica-gel column: 1,000 mm), eluting with a gradient of CH_2_Cl_2_/MeOH (100:0, 100:1, 100:2, 100:4, 100:8, 100:16, v/v), to yield six fractions (Frs. 1–6). Fr6 was recrystallized from methanol, yielding a white crystal (compound **5**, 3 mg). Fr1 and Fr2 were further purified by crystallization from CH_2_Cl_2_/MeOH to give the compounds **1** (7 mg) and **2** (3 mg), respectively. Fr4 was subjected to a Sephadex LH-20 column with MeOH to yield compounds **3** (5 mg) and **4** (2 mg).

The structures of all compounds were determined using spectroscopic analysis. High Resolution-Mass Spectrometry (HR-ESI-MS) spectra were measured on a TripeTOF 4600 mass analyzer (Bruker, United States). ^1^H/^13^C-nuclear magnetic resonance (NMR) data were acquired using Agilent DD2 600Hz spectrometer (Agilent, United States) with tetramethylsilane (TMS) as an internal standard, and chemical shifts (δ) were reported as parts per million (ppm) values.

### Antibacterial assay of compounds

The antibacterial activity of the pure compounds isolated from QTH17 were determined using previously described the method of filter paper dispersion (Xu et al., 2020) and the microdilution method of the minimum inhibitory concentrations (MICs) (Li et al., 2014). MICs of compounds were measured and determined at five different concentration gradients in disposable 96-well microtiter dishes. A stock solution of each compound (200 μg/mL) was further twofold diluted in LB broth and added separately into individual microplate wells (100 μL/well). Then, a standard amount of the tested microbes (1.0 × 10^6^ CFU/mL) were added per well. Thus, the compounds were tested in the final concentration range of 100–3.125 μg/mL. The 96-well plates were incubated at 37°C for 12–14 h. The lowest concentration of compounds at which the bacterial growth was inhibited, was defined as MIC, as shown by no turbidity. Gentamicin sulfate was used as a positive control. Each determination was performed three times.

## Results

### Diversity of cultivable fungi

**FIGURE 1:**
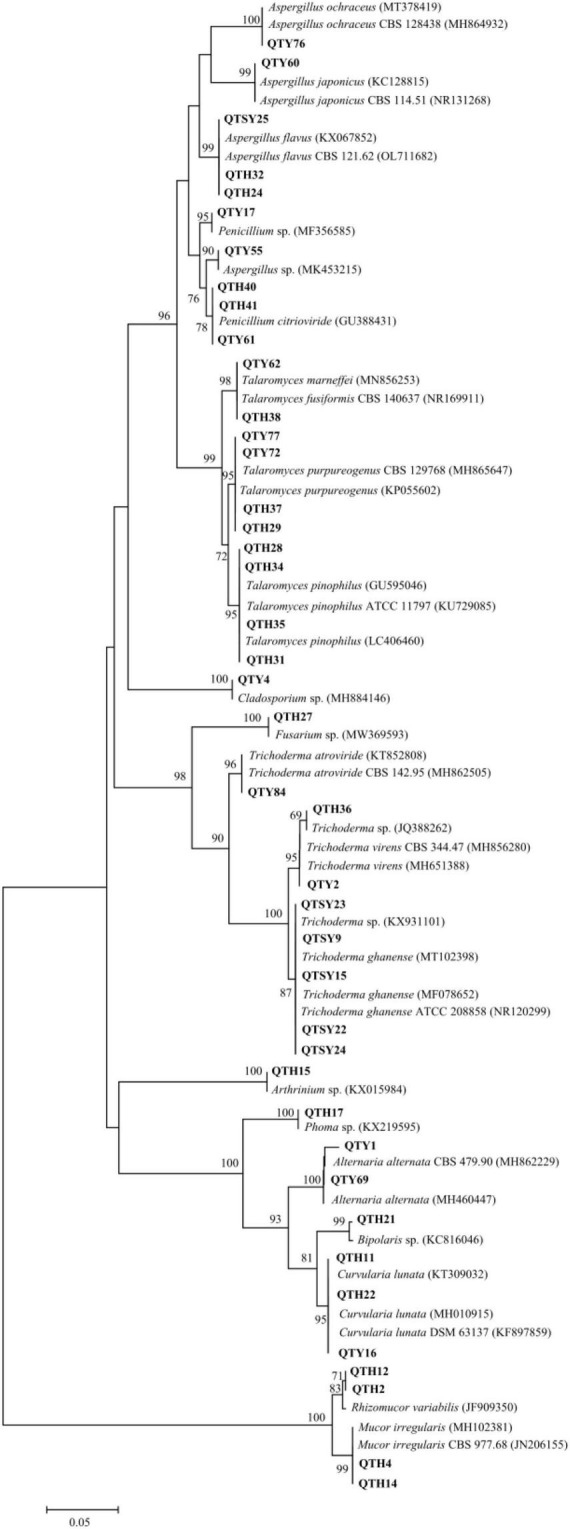
Neighbor-joining phylogenetic tree of ITS rDNA sequences of gut-associated fungi.

In this study, a total of 42 fungi were isolated from the guts of *C. Servilia* using the culture-dependent method (Table 1 and Figure 1). All fungal isolates were identified by the ITS1/IST4 region of the 5.8S rDNA gene and comparison with available sequences in GenBank. Sequence analysis revealed that all fungal isolates were classified into 2 fungal phyla, 4 classes, 6 orders, 9 families, 13 genera. Ascomycota was the predominant fungi with a proportion of 90.48%. Thirty-eight isolates were grouped into three classes [Eurotiomycetes (47.62%), Sordariomycetes (23.81%), and Dothideomycetes (19.05%)] within the phylum Ascomycota. The other 4 strains (9.52%) were distributed in the Zygomycete within the phylum Zygomycota.

**TABLE 1:** Phylogenetic analysis of cultivable fungi associated with gut of *C. Servilia*.

Isolate code	Closest match	Accession no.	Coverage/max ident	GenBank no.
QTH36	*Trichoderma* sp.	JQ388262	99/99	ON248244
QTSY23	*Trichoderma* sp.	KX931101	99/100	ON248245
QTY84	*Trichoderma atroviride*	KT852808	99/99	ON248246
QTSY9	*Trichoderma ghanense*	MT102398	99/99	ON248247
QTSY15	*Trichoderma ghanense*	MF078652	99/99	ON248248
QTSY22	*Trichoderma ghanense*	MF078652	99/99	ON248249
QTSY24	*Trichoderma ghanense*	MF078652	99/100	ON248250
QTY2	*Trichoderma virens*	MH651388	99/99	ON248251
QTH11	*Curvularia lunata*	KT309032	99/99	ON248252
QTH22	*Curvularia lunata*	MH010915	99/100	ON248253
QTY16	*Curvularia lunata*	KT309032	99/99	ON248254
QTH27	*Fusarium* sp.	MW369593	99/99	ON248255
QTH21	*Bipolaris* sp.	KC816046	99/99	ON248256
QTH15	*Arthrinium* sp.	KX015984	99/99	ON248257
QTY4	*Cladosporium* sp.	MH884146	100/99	ON248258
QTH17	*Phoma* sp.	KX219595	97/99	ON248259
QTH12	*Rhizomucor variabilis*	JF909350	92/99	ON248260
QTH2	*Rhizomucor variabilis*	JF909350	92/99	ON248261
QTH4	*Mucor irregularis*	MH102381	98/99	ON248262
QTH14	*Mucor irregularis*	MH102381	99/99	ON248263
*QTY1	*Alternaria alternata*	MH460447	98/98	ON248264
QTY69	*Alternaria alternata*	MH862229	99/99	ON248265
QTY17	*Penicillium* sp.	MF356585	100/99	ON248266
QTH40	*Penicillium citrioviride*	GU388431	99/100	ON248267
QTH41	*Penicillium citrioviride*	GU388431	100/99	ON248268
QTY61	*Penicillium citrioviride*	GU388431	98/99	ON248269
QTY55	*Aspergillus* sp.	MK453215	99/100	ON248270
QTSY25	*Aspergillus flavus*	KX067852	99/99	ON248271
QTH32	*Aspergillus flavus*	KX067852	98/99	ON248272
QTH24	*Aspergillus flavus*	KX067852	98/99	ON248275
QTY76	*Aspergillus ochraceus*	MT378419	99/100	ON248273
QTY60	*Aspergillus japonicus*	KC128815	99/100	ON248274
QTH38	*Talaromyces fusiformis*	NR169911	99/99	ON248276
QTY62	*Talaromyces marneffei*	MN856253	100/99	ON248277
QTH28	*Talaromyces pinophilus*	GU595046	99/99	ON248278
*QTH31	*Talaromyces pinophilus*	GU595046	99/98	ON248284
QTH34	*Talaromyces pinophilus*	GU595046	99/99	ON248279
QTH35	*Talaromyces pinophilus*	LC406460	100/99	ON248280
QTH29	*Talaromyces purpureogenus*	KP055602	98/99	ON248281
QTH37	*Talaromyces purpureogenus*	KP055602	98/99	ON248282
QTY77	*Talaromyces purpureogenus*	KP055602	97/99	ON248283
QTY72	*Talaromyces purpureogenus*	KP055602	97/99	ON248285

*Potentially new species.

The largest number (20) of the strains belonged to the class Eurotiomycetes and was only distributed in Eurotiales order. The most common genera identified was *Talaromyces* in this study. In total, 10 strains were in the genus *Talaromyces* and identified as *T. fusiformis* (1 isolate), *T. marneffei* (1 isolate), *T. pinophilus* (4 isolates), and *T. purpureogenus* (4 isolates). Among them, the strain QTH31 showed only 98% similarity to *T. pinophilus*, which suggested a potential new species. Six isolates of the family Aspergillaceae were mainly concentrated in the genus *Aspergillus*. Three strains showed highly similar to *A. flavus* with an identity of 99%. Another three strains belonged to the genera *Aspergillus* and had 100% similar sequences to *Aspergillus* sp., *A. japonicus*, and *A. ochraceus*, respectively. The last four strains belonged to the genus *Penicillium*, and three strains of them were identified as *P. citrioviride*.

Another representative class was Sordariomycetes, including Hypocreales, and Xylariales orders. Eight strains were in the genus *Trichoderma* and were identified as *Trichoderma* sp. (2 isolates), *T. atroviride* (1 isolate), *T. ghanense* (4 isolates), and *T. virens* (1 isolate). The other one strain belonging to the family Hypocreaceae was identified as *Fusarium* sp. with an identity of 99%. The last one strain belonging to *Arthrinium* genus showed similar to *Arthrinium* sp. with an identity of 99%.

The strains of Dothideomycetes (8 isolates) were assigned to two orders, including the Pleosporales (7 isolates) and Cladosporiales (1 isolate). The three strains in Pleosporaceae showed highly similar to *Curvularia lunata* with more than 99% identity. Both strains belonging to Pleosporaceae were identified as *Alternaria alternata* with more than 99% identity. The strain QTY1 showed only 98% similarity to *A. alternata*, which was regarded as a potential new species. In addition, two strains of Pleosporales were identified as *Bipolaris* sp. and *Phoma* sp., respectively. Only one strain belonging to the order Cladosporiales was grouped into the genus *Cladosporium* with an identity of 99% match to *Cladosporium* sp.

Finally, the last four strains of class Zygomycete belonging to the phylum Zygomycota were distributed in the same order Mucorales. Both strains belonging to Mucoraceae were identified as *Rhizomucor variabilis* with an identity of 99%. The other two strains exhibited a sequence match of 99% to *Mucor irregularis.*

### Culture-independent community analysis

**FIGURE 2:**
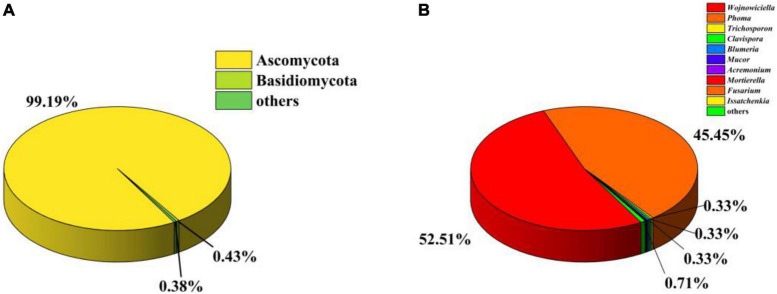
Analysis of culture-independent microbial communities. Relative abundance of phylum **(A)** and genus **(B)** within gut fungal communities.

To investigate the gut fungal community composition of *C. Servilia*, the culture-independent method was conducted. In total, 244,994 final reads with an average length of 290 bp were obtained from three samples, and 376 OTUs were acquired (QTC1, QTC2, QTC3) (Supplementary Table 1). Rarefaction curves showed that all samples reached stable values, indicating that the sequencing depth was sufficient (Supplementary Figure 1). In terms of alpha diversity, all samples displayed similar levels of diversity, including the Ace, Chao1, Shannon, and Simpson diversity indexes (Supplementary Table 1). The fungal community composition of different samples was estimated at the phylum and genus levels. The OTUs from all three samples were primarily assigned to the phyla Ascomycota (99.19%) and Basidiomycota (0.43%), and Ascomycota was the most abundant phylum (Figure 2A). At the genus level, 136 genera were identified across investigated gut samples (Supplementary Table 2). Among which, *Wojnowiciella* (52.51%) and *Phoma* (45.45%) were the main dominant fungal genera in gut fungi (Figure 2B). Moreover, several genii of cultivable filamentous fungi were also found in the culture-independent method, such as the genus *Trichoderma*, *Curvularia*, *Fusarium*, *Bipolaris*, *Cladosporium*, *Rhizomucor*, *Mucor*, *Alternaria*, *Penicillium*, *Aspergillus*, and *Talaromyces*, etc. (Supplementary Table 2).

### Antibacterial activities of cultivable fungi

The antibacterial results of crude extracts of representative strains from 11 different genera were shown in Table 2. The results showed that five fungal crude extracts (QTH14, QTH15, QTH17, QTH21, and QTH27) exhibited inhibitory activities against at least one of the bacterial strains under the concentration of 30 μg/filter paper. Among them, QTH17 displayed potent antibacterial activities against *E. coli*, *M. tetragenus* and *S. aureus* with the disc diameter of IZD of 6.50, 10.80, and 8.70 mm, respectively, which were slightly weaker than those of the positive gentamicin sulfate with the IZD of 13.60, 16.50, and 12.30 mm, respectively.

**TABLE 2:** Inhibition zone diameters of inhibiting effects of fungal extracts on three tested bacteria (mm).

Proposed identity	Isolate code	*E. coli*	*M. tetragenus*	*S. aureus*
*Mucor irregularis*	QTH14	NI	7.20 ± 0.47	NI
*Arthrinium* sp.	QTH15	NI	8.30 ± 0.82	NI
*Phoma* sp.	QTH17	6.50 ± 0.47	10.80 ± 0.00	8.70 ± 0.47
*Bipolaris* sp.	QTH21	NI	9.20 ± 0.24	8.20 ± 0.47
*Talaromyces pinophilus*	QTH31	NI	NI	NI
*Aspergillus flavus*	QTH32	NI	NI	NI
*Trichoderma* sp.	QTH36	NI	NI	NI
*Penicillium citrioviride*	QTH41	NI	NI	NI
*Alternaria alternata*	QTY1	NI	NI	NI
*Cladosporium* sp.	QTY4	NI	NI	NI
*Fusarium* sp.	QTH27	7.00 ± 0.00	6.50 ± 0.00	NI
Gentamycin sulfate^a^		13.60 ± 0.47	16.50 ± 0.82	12.30 ± 0.47

^a^Gentamycin sulfate as the positive control. “NI” means not inhibited; the concentration for the test is 30 μg/filter paper.

### Separation and identification of the compounds of QTH17

**FIGURE 3:**
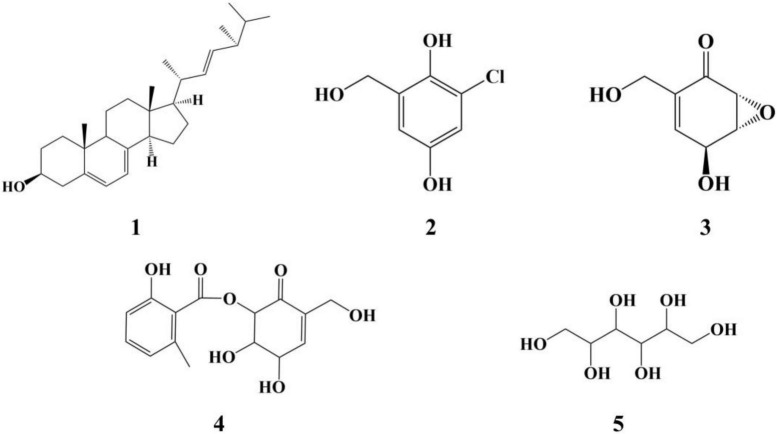
The structure of compounds **1–5**.

Five compounds were purified from the ME liquid fermentation product of *Phoma* sp. QTH17 (Figure 3), and their structures were determined to be ergosterol **(1)** (Huang et al., 2011), 3-chlorogentisyl alcohol **(2)** (Li et al., 2005), epoxydon **(3)** (Venkatasubbaiah et al., 1992a), epoxydon 6-methylsalicylate ester **(4)** (Venkatasubbaiah and Chilton, 1992b), and mannitol **(5)** (Branco et al., 2010) by spectroscopic data (Supplementary Figures 2–9) analyses, and the comparison of their derivative data in the literature.

### Antibacterial activities of compounds

The antibacterial activities of the compounds isolated from QTH17 were shown in Table 3. The results revealed that three compounds (**2**–**4)** exhibited inhibitory activities against at least one of the bacterial strains under the concentration of 30 μg/filter paper. Specifically, compound **3** exhibited potent antibacterial activities against *E. coli*, *M. tetragenus*, and *S. aureus* with the IZD of 7.00, 14.00, and 12.50 mm, respectively, which were slightly weaker than those of the positive gentamicin sulfate with the IZD of 11.13, 18.30, and 12.13 mm. Compound **2** exhibited moderate inhibitory activities against *M. tetragenus* with the IZD of 9.00 mm, while it had no inhibition effect on *E. coli* and *S. aureus*. Compound **4** only displayed potent inhibitory activity against *S. aureus* with the IZD of 11.00 mm. However, compounds **3** and **4** lacked activities against three tested strains with MIC values of more than 100 μg/mL.

**TABLE 3:** Inhibition zone diameter (mm) and minimum inhibitory concentration (MIC) values (μg/mL) of compounds against three tested bacteria.

Compounds	*E. coli*		*M. tetragenus*		*S. aureus*	
			
	IZD^a^	MIC	IZD^a^	MIC	IZD^a^	MIC
2	NI	NT	9.00 ± 0.05	NT	NI	NT
3	7.00 ± 0.03	>100	14.00 ± 0.24	>100	12.50 ± 0.20	>100
4	NI	>100	NI	>100	11.00 ± 0.23	>100
Gentamycin sulfate^b^	11.13 ± 0.20	>100	18.30 ± 0.31	12.5	12.13 ± 0.58	6.25

^a^Results are presented as the mean ± standard; “NI” means not inhibited; the concentration for the test is 30 μg/filter paper; “NT” means not tested. ^b^Gentamycin sulfate as the positive control.

## Discussion

The insect gut contains a vast number of microbes, including bacteria, fungi, and viruses, many of which influence the health and development of their hosts (Muratore et al., 2020). However, the fungal component of the microbiota is frequently overlooked compared to bacteria (Engel and Moran, 2013). Insect-associated fungi have been an essential source of new microbial resources and bioactive compounds (Zhang et al., 2011; Nagam et al., 2021; Tong et al., 2021). Culture-dependent and -independent methods are commonly used to investigate the fungal community composition of insect gut (Xu et al., 2020; Callegari et al., 2021). Forty-two fungi, including two potentially new species, were isolated and characterized by the culture-dependent method and molecular biological identification in this study. Concomitantly, the fungal community composition of the gut was further analyzed by the culture-independent method. Furthermore, five compounds were purified and characterized from *Phoma* sp. QTH17 has antibacterial activities. To our knowledge, this is the first report on understanding fungal community composition and screening of biologically active secondary metabolites of gut-associated fungi isolated from the *C. Servilia*, which will provide a resource for microbial diversity and antibiotics.

Thirteen different genera were found in the guts of *C. Servilia* by the culture-dependent method, which was highly similar to the gut-associated fungi of *Pantala flavescens* larvae (Shao et al., 2015). Moreover, *Penicillium*, *Aspergillus*, and *Cladosporium* have been associated with other insects’ guts, such as termites, honeybees, beetles, and palm weevils (Moubasher et al., 2017; Xu et al., 2020). *Trichoderma*, *Cladosporium*, *Penicillium*, and *Talaromyces* were also found in the guts of aquatic insect larvae, which have cellulolytic potential (Belmont-Montefusco et al., 2020). Besides, *C. lunata* and *A. alternata* isolated from the gut have been reported as plant pathogens (Fahim Abbas et al., 2021). However, some culturable fungi are the first reported fungi of dragonfly gut, such as *Bipolaris* sp., *Arthrinium* sp., *M. irregularis*, *T. marneffei*, *T. pinophilus*, and *T. purpureogenus*. This study further confirmed the association between filamentous fungi and dragonflies.

However, isolation methods of culturable fungi still have some limitations. The composition of *C. Servilia* gut fungal community was accurately evaluated by the culture-independent method in this study. A total of 136 genus-level OTUs were detected in the dragonfly gut sample, whereas only thirteen genera were isolated by culture. This result provides the impetus for strategies to obtain more fungi from the dragonfly gut in future studies, for instance, media-fungus pairings (Oberhardt et al., 2015). Notably, the most frequently isolated cultivable fungi correspond with fungal OTUs in the culture-independent method. Nevertheless, only *Phoma* sp. was isolated from the main dominant fungal genera, *Wojnowiciella* was not isolated. Synthetic low-nutrient agar (SNA) medium at 25°C was used to isolate *Wojnowiciella*, which remains to be explored further (Marin-Felix et al., 2019). Additionally, due to the taxonomic resolution of fungal community has remained relatively low, some fungal OTUs were unidentified (Muratore et al., 2020).

To validate that gut-associated fungi represent a source of antimicrobials with activity against pathogenic bacteria, 11 different genera were tested *in vitro* assays using three different bacteria. However, there are only a few strains that have shown activity against pathogenic bacteria. For example, *Fusarium* sp. QTH27, *Mucor irregularis* QTH14, and *Bipolaris* sp. QTH21 exhibited weak antibacterial activity against *M. tetragenus*. Similarly, most fungi isolated from the gut of *Pantala flavescens* larvae had weak or no activity against pathogenic bacteria (Shao et al., 2015). Notably, *Phoma* sp. QTH17 has shown higher activities against both Gram-positive and Gram-negative bacteria. Moreover, *Phoma* spp. was a significant source of bioactive compounds, which have demonstrated a range of antibacterial, antifungal, and cytotoxic activity (Rai et al., 2020). Ergosterol **(1)** and mannitol **(5)** are common fungal metabolites and act as fungal cell membrane growth factors and metabolic stores, respectively (de Lira Mota et al., 2012; Meena et al., 2015). 3-Chlorogentisyl alcohol **(2)**, epoxydon **(3)**, and epoxydon 6-methylsalicylate ester **(4)** have been isolated from *Phoma* spp. (Venkatasubbaiah et al., 1992a; Nenkep et al., 2010).

The antibacterial bioassay showed that compound **3** showed the moderate antibacterial activities against *E. coli*, *M. tetragenus*, and *S. aureus* using the filter paper dispersion method, but no inhibition was found in the MIC test with the maximum concentration of 100 μg/mL. The result was consistent with previous report that compound **3** gave the MIC value of 128 μg/mL against *S. aureus* (Trisuwan et al., 2008). Additionally, three bacterial strains were not susceptible to compound **4** using the microdilution method. Therefore, MIC values might better mirror the antibacterial activity of compound against the tested strains (Di Lodovico et al., 2020).

## Conclusion

Here, the diversity of the gut-associated fungi isolated from the *C. Servilia* was analyzed using both culture-dependent and culture-independent methods. This study improved our understanding of gut-associated fungi and further increase fungal species from *C. Servilia*. Antibacterial activity assays showed that several of these fungi have antibacterial activities, among which the fungus *Phoma* sp. QTH17 was the most prominent. In addition, five known compounds were identified. These results suggest that dragonfly gut-associated fungi represent a promising and underexplored resource for exploring antibiotics.

## Data availability statement

The datasets presented in this study can be found in online repositories. The names of the repository/repositories and accession number(s) can be found below: https://www.ncbi.nlm.nih.gov/ (ON248244–ON248285 and PRJNA827522).

## Author contributions

YZ designed the research and supervised the study. PC, LL, SS, ZH, and KK performed the experiments and analyzed the data. PC and LL analyzed and wrote the manuscript. All authors revised the manuscript and approved the final version for submission.
