# Supplementary material for: Diversity, antibacterial activity and chemical analyses of gut-associated fungi isolated from the Crocothemis servilia
Source: Front Microbiol. 2022 Sep 16;13:970990. doi: 10.3389/fmicb.2022.970990 (PMC9523248; doi:10.3389/fmicb.2022.970990)
Supplement: Supplementary file 1 [file Data_Sheet_1.docx]

Diversity, Antibacterial Activity and Chemical Analyses of Gut-Associated Fungi Isolated from the *Crocothemis Servilia*

Pu Cui^1,†^, Lijun Liu^1,†^, Zhongdi Huang^1^, Shuping Shi^1^, Kun Kong^1^ and Yinglao Zhang^1,^ ^2*^

^1^School of Life Sciences, Anhui Agricultural University, Hefei 230036, China

^2^State Key Laboratory of Tea Plant Biology and Utilization, Anhui Agricultural University, Hefei, China

^*^Correspondence:

Yinglao Zhang

zhangyl@ahau.edu.cn;

^†^These authors contributed equally to the study and share first authorship.

**Contents:**

**NMR and ESI-MS data for compounds 1-5.**

**Supplementary Tables**

**Table S1** The unique tags and a diversity of gut-associated fungi from *Crocothemis Servilia*.

**Table S2** Taxonomic assignment of the OTUs obtained in the study and their relative abundance at each *Crocothemis Servilia* gut sample.

**Supplementary Figures**

**Figure S1** Rarefaction curves of amplicon sequencing for all samples.

**Figure S2** The ^1^H NMR spectrum of **1** in CDCl_3_ (600 MHz).

**Figure S3** The ^1^H NMR spectrum of **2** in acetone-*d*_6_ (600 MHz).

**Figure S4** The ^13^C NMR spectrum of **2** in acetone-*d*_6_ (150 MHz).

**Figure S5** The ^1^H NMR spectrum of **3** in CDCl_3_ (600 MHz).

**Figure S6** The ^13^C NMR spectrum of **3** in CDCl_3_ (150 MHz).

**Figure S7** The ^1^H NMR spectrum of **4** in DMSO-*d*_6_ (600 MHz).

**Figure S8** The ^13^C NMR spectrum of **4** in DMSO-*d*_6_ (150 MHz).

**Figure S9** The ^1^H NMR spectrum of **5** in DMSO-*d*_6_ (600 MHz).

**NMR and ESI-MS data for compounds 1-5.**

Ergosterol (**1**): white powder; ESI-MS: m/z: 395 [M-H]^-^, calculated for C_28_H_44_O, 396; ^1^H NMR (600 MHz, CDCl_3_) *δ*: 5.57 (1H, dd 5.4, H-6), 5.38 (1H, d 2.9, H-7), 5.20 (1H, dd , H-23), 5.19 (1H, dd, H-22), 3.64 (1H, m, H-3), 1.04 (3H, d, H-21), 0.94 (3H, s, H-19), 0.92 (3H, d, H-28), 0.83 (6H, d, H-26/H-27), 0.63 (3H, s, H-18).

3-Chlorogentisyl alcohol (**2**): red solid; ESI-MS: m/z: 173 [M-H]^-^, calculated for C_7_H_7_ClO_3_, 174; ^1^H NMR (600 MHz, acetone-*d*_6_), *δ*: 6.73 (1H, d, *J* = 2.4 Hz, H-4), 6.77 (1H, d, *J* = 2.4 Hz, H-6), 4.70 (2H, s, H2-7); ^13^C NMR (150 MHz, acetone-*d*_6_), *δ*: 121.1 (s, C-1), 151.5 (s, C-2), 131.3 (s, C-3), 115.2 (d, C-4), 144.6 (s, C-5), 114.2 (d, C-6), 61.9 (t, C-7).

Epoxydon (**3**): rufous oily; ESI-MS: m/z: 155 [M-H]^-^, calculated for C_7_H_8_O_4_, 156; ^1^H NMR (600 MHz, CDCl_3_), *δ*:6.52 (1H, d, *J* = 1.77 Hz, H-3), 4.73 (1H, q, *J* = 0.34, 0.86 Hz, H-4), 3.87 (1H, t, *J* = 0.86,0.34 Hz, H-5), 3.53 (1H, d, *J* = 0.34 Hz, H-6), 4.35 (2H, dd, H-7); ^13^C-NMR (150 MHz, CDCl_3_), *δ*: 193.9(C-1), 134.8(C-2), 140.8(C-3), 65.2(C-4), 54.6(C-5), 54.1(C-6), 60.8 (C-7).

Epoxydon 6-methylsalicylate ester (**4**): white crystal; ESI-MS: m/z: 331 [M+Na]^+^, calculated for C_15_H_16_O_7_, 308; ^1^H NMR (600 MHz, DMSO-*d*_6_), *δ*:9.86 (1H, s, 2-OH), 2.37 (3H, s, 7-H), 6.74 (1H, d, *J* = 8.0 Hz, 5-H), 7.19 (1H, t, *J* = 8.0 Hz, 4-H), 6.72 (1H, d, *J* = 8.0 Hz, 3-H), 4.10 (2H, m, 7-H), 5.67(1H, d, *J* = 10.5 Hz, 6-H), 3.98 (1H, dd, *J* =10.4, 3.8 Hz, 5-H), 4.41 (1H, m, 4-H), 6.93 (1H, d, *J* = 5.7 Hz, 3-H); ^13^C NMR (150 MHz, DMSO-*d*_6_), *δ*:194.1 (C-1), 143.2 (C-2), 139.6 (C-3), 66.7 (C-4), 70.7 (C-5), 77.2 (C-6), 58.6 (C-7), 120.5 (C-1´), 157.4 (C-2´), 115.0 (C-3´), 132.7 (C-4´), 122.4 (C-5´), 138.9 (C-6´), 21.5 (C-7´), 168.8 (C-8´).

Mannitol (**5**): white crystal; ESI-MS: m/z: 181 [M-H]^-^, calculated for C_6_H_14_O_6_, 182; ^1^H NMR (600 MHz, DMSO-*d*_6_), *δ*: 4.33 (d, *J* = 5.0 Hz, 2H), 4. 25 (t, *J* = 6.0 Hz, 2H), 4. 06 (d, *J* = 7.0 Hz, 2H), 3.55(m, 2H), 3.54 (t, *J* = 7.5 Hz, 2H), 3.48 (m, 2H), 3.40 (m, 2H).

**Supplementary Tables**

**Table S1** The unique tags and a diversity of gut-associated fungi from *Crocothemis Servilia*.

| Sample | Sequence reads | | Rarefied alpha diversity | | | | |
| --- | --- | --- | --- | --- | --- | --- | --- |
|  | Total sequence | Total OTUs | Ace | Chao1 | Shannon | Simpson | Good's Coverage |
| QTC1 | 78053 | 147 | 147.76 | 147.02 | 2.22 | 0.73 | 1.000 |
| QTC2 | 85209 | 129 | 141.46 | 136.00 | 2.11 | 0.72 | 1.000 |
| QTC3 | 81732 | 105 | 118.02 | 115.50 | 0.75 | 0.23 | 1.000 |

**Supplementary Figures**


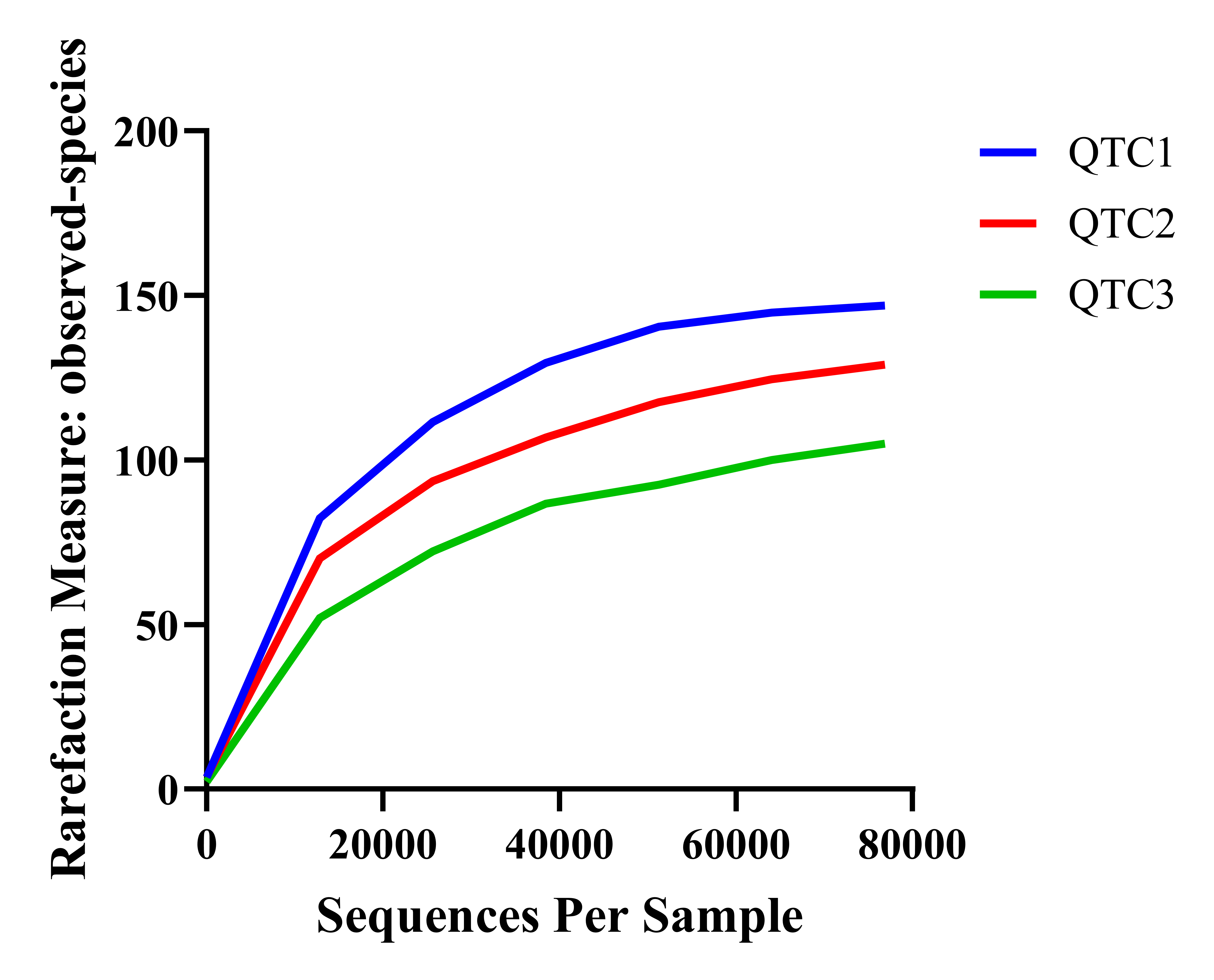


**Figure S1** Rarefaction curves of amplicon sequencing for all samples.

**
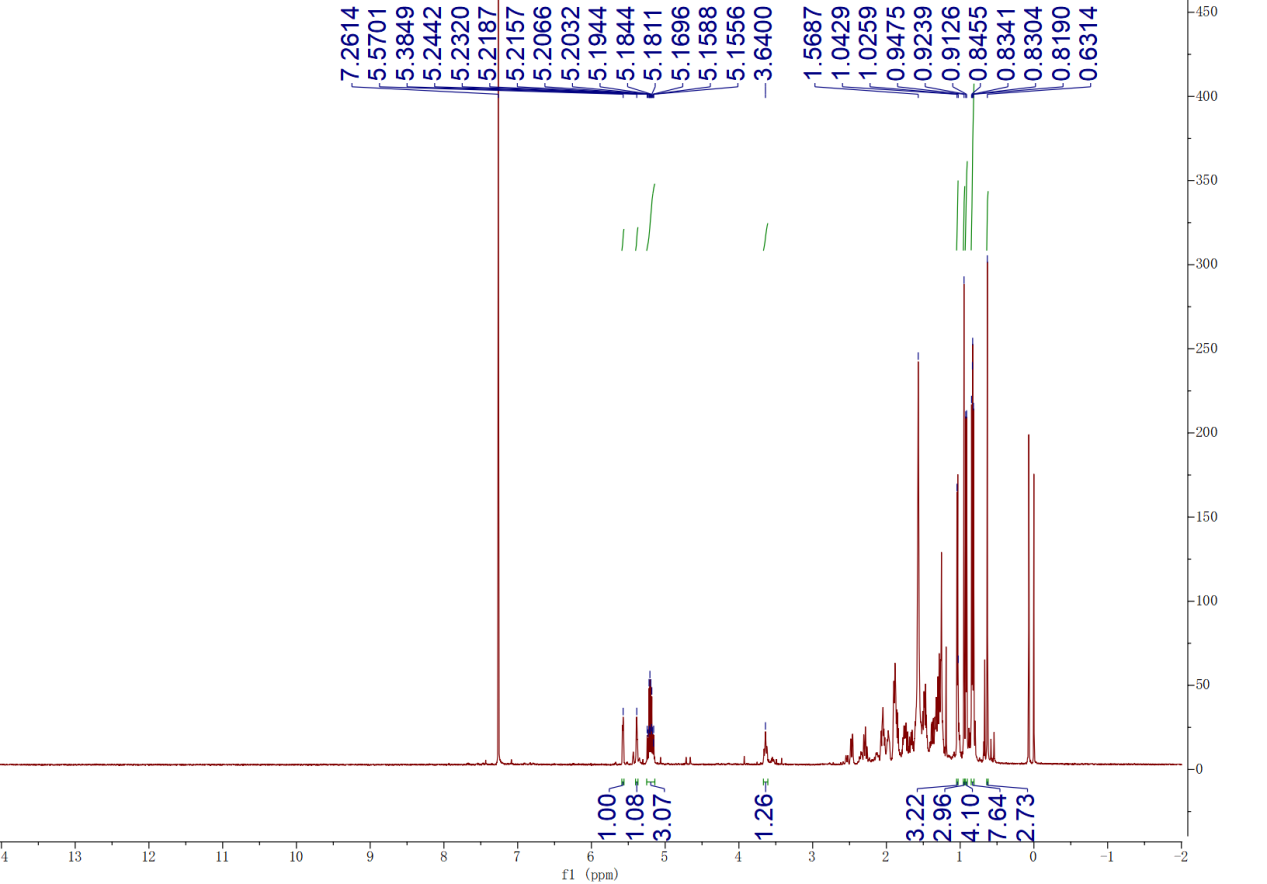
**

**Figure S2** The ^1^H NMR spectrum of **1** in CDCl_3_ (600 MHz).

**
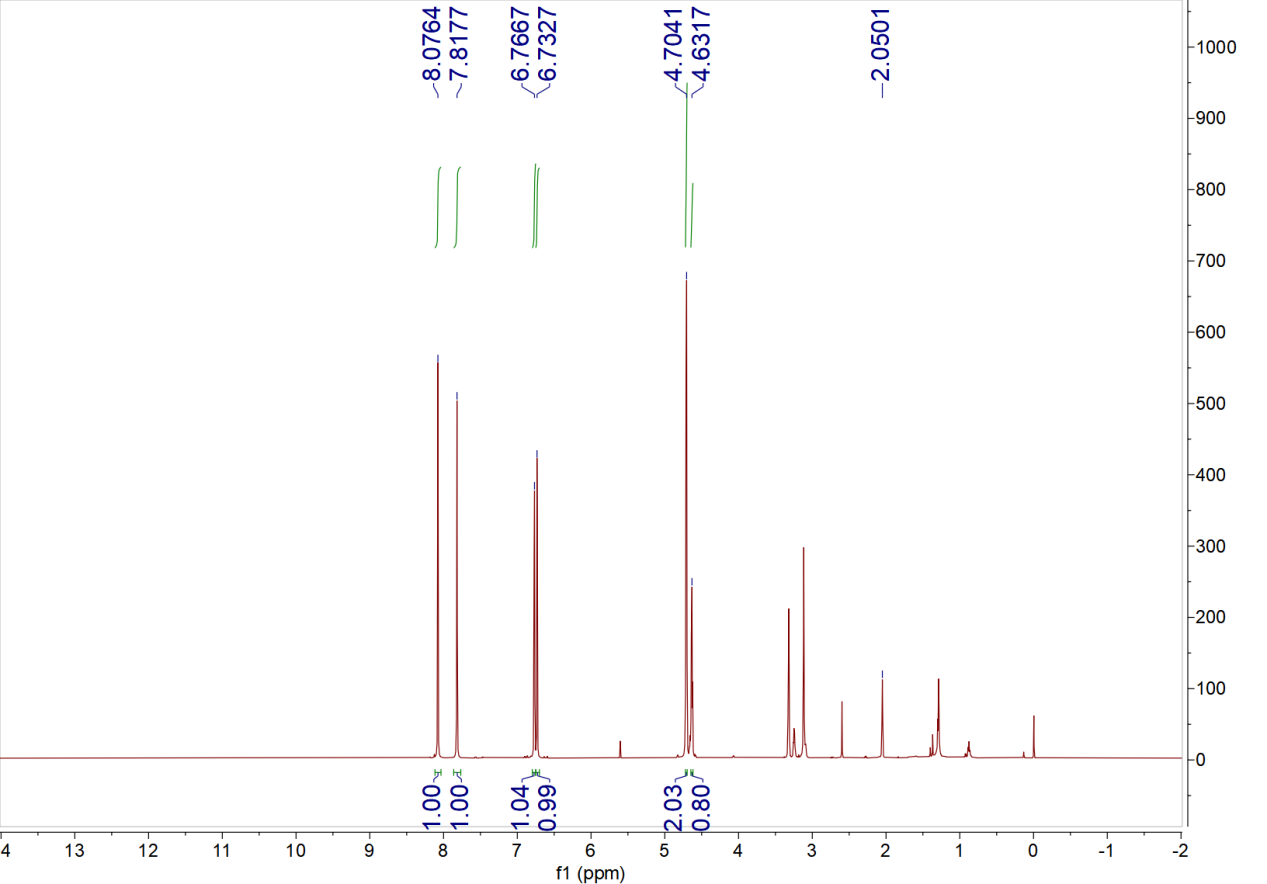
**

**Figure S3** The ^1^H NMR spectrum of **2** in acetone-*d*_6_ (600 MHz).


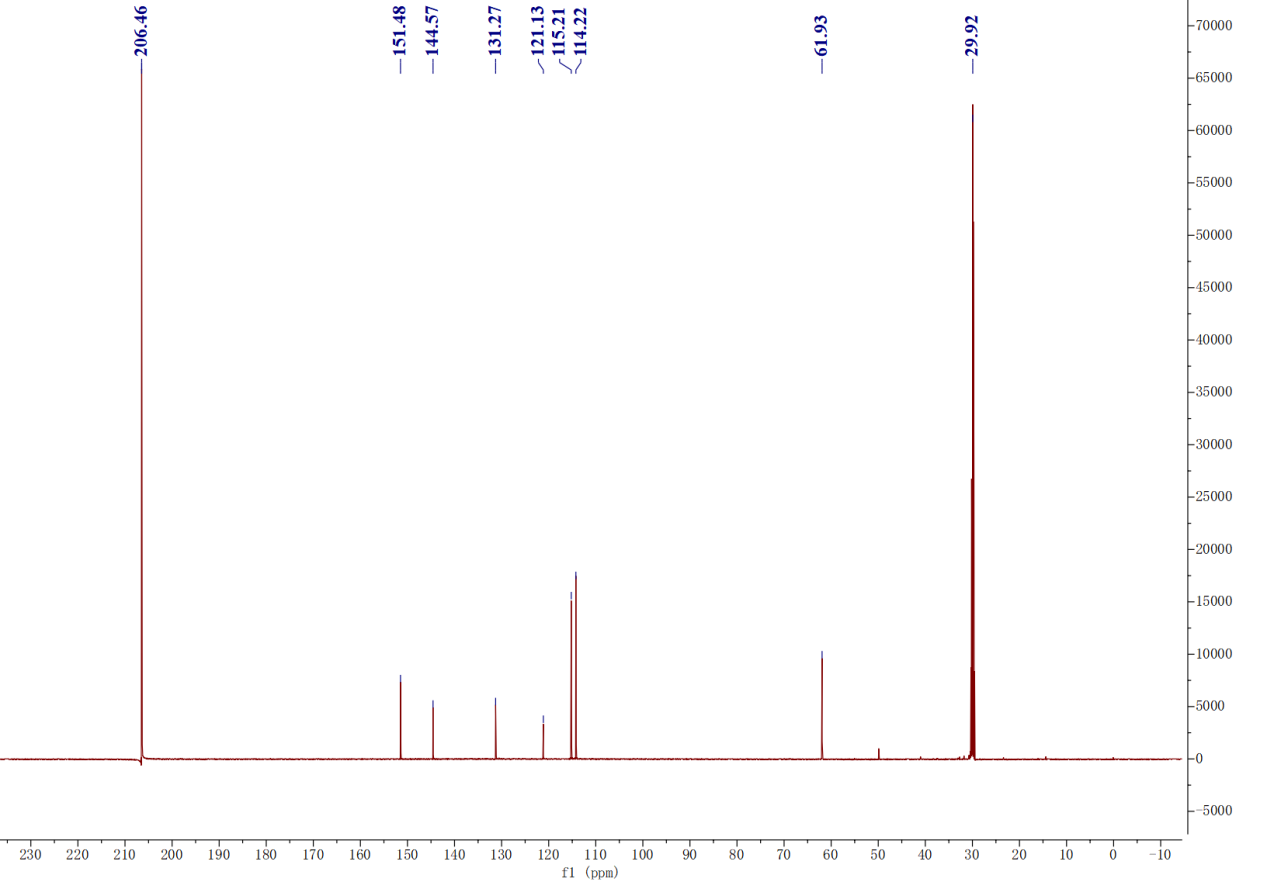


**Figure S4** The ^13^C NMR spectrum of **2** in acetone-*d*_6_ (150 MHz). **
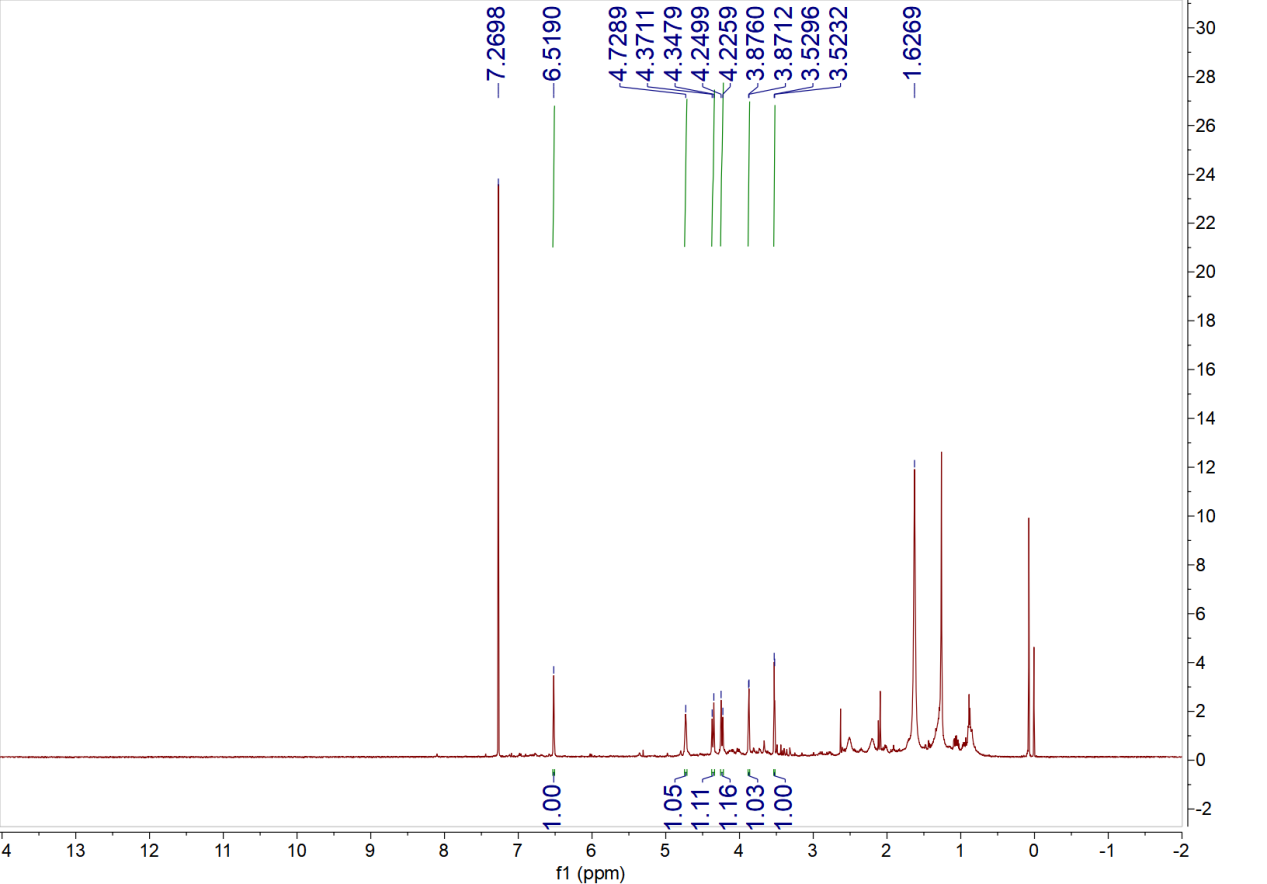
**

**Figure S5** The ^1^H NMR spectrum of **3** in CDCl_3_ (600 MHz).


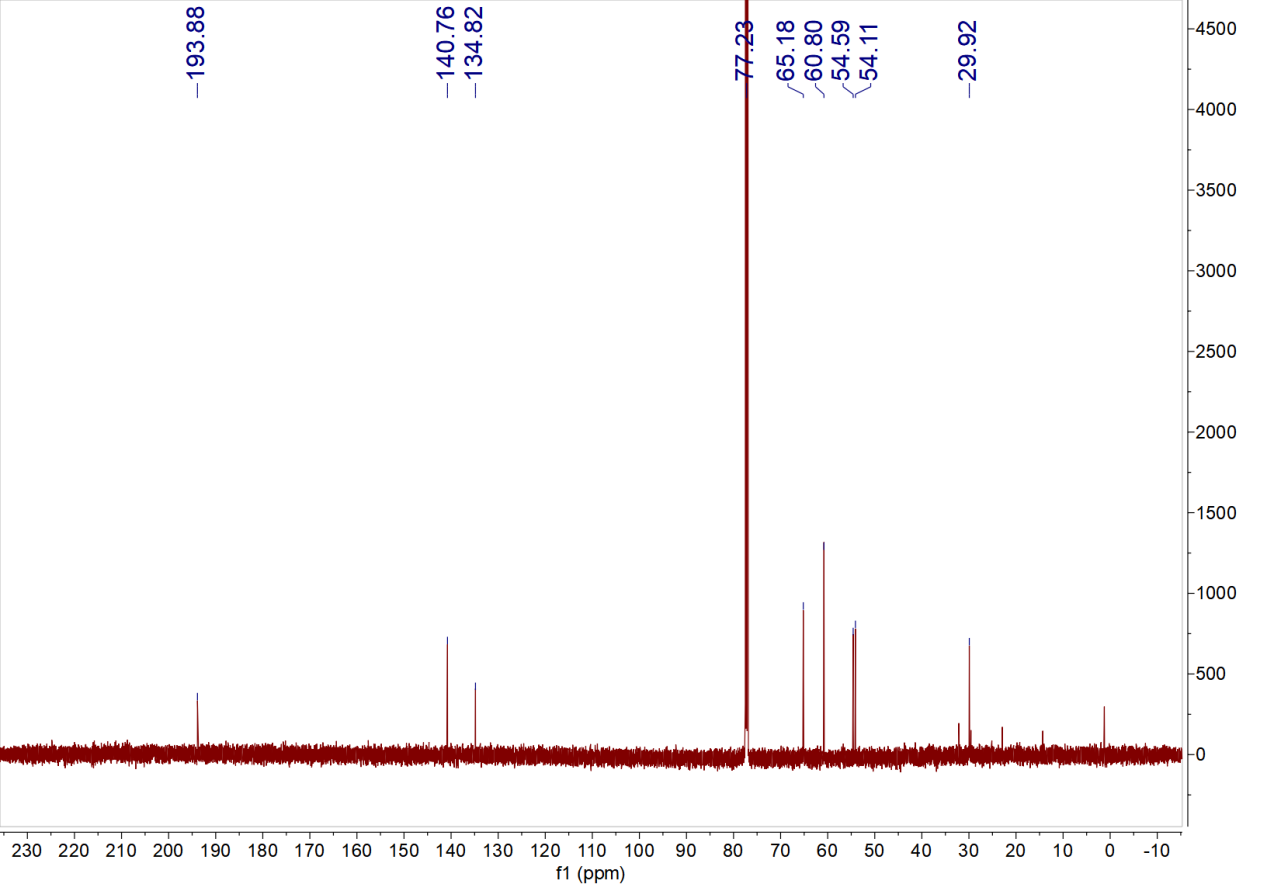


**Figure S6** The ^13^C NMR spectrum of **3** in CDCl_3_ (150 MHz).


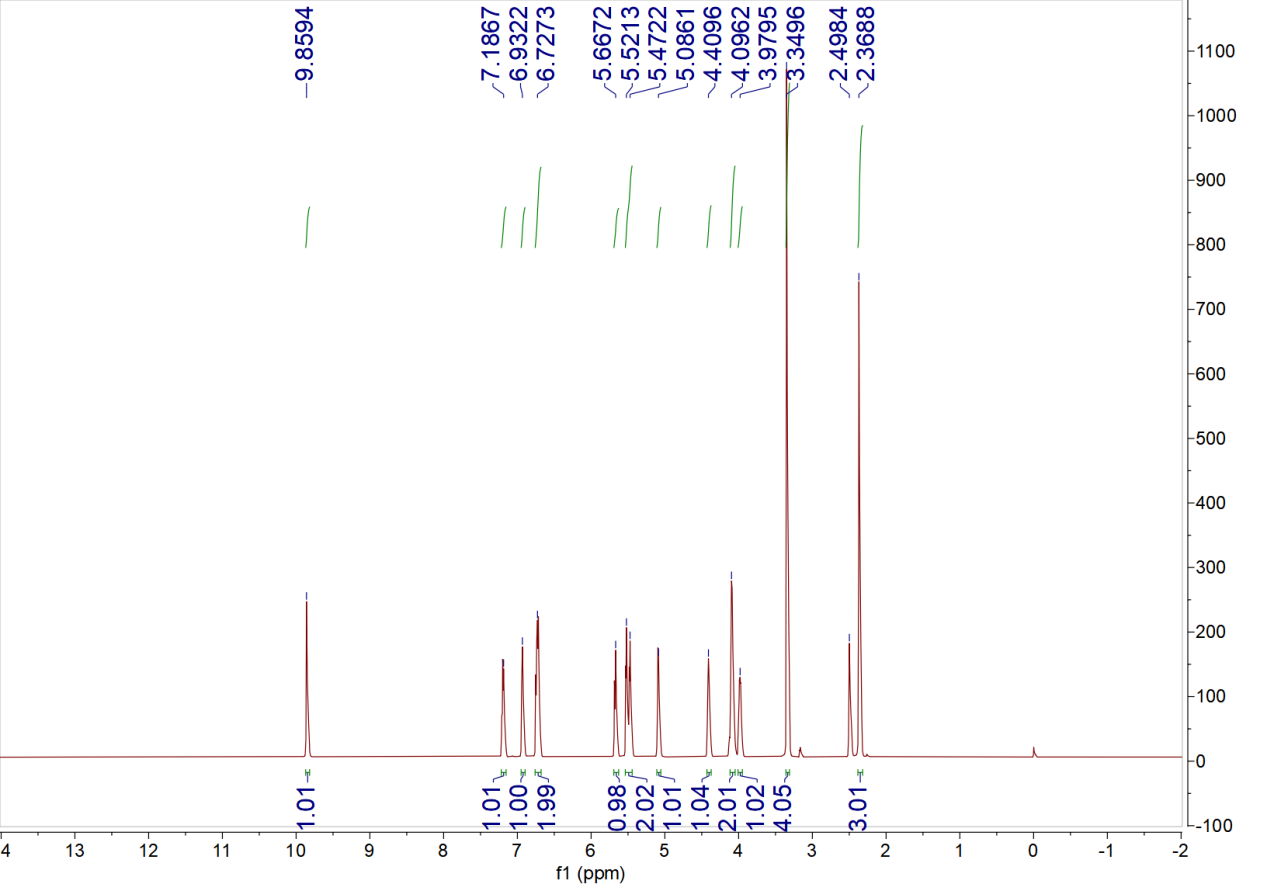


**Figure S7** The ^1^H NMR spectrum of **4** in DMSO-*d*_6_ (600 MHz).


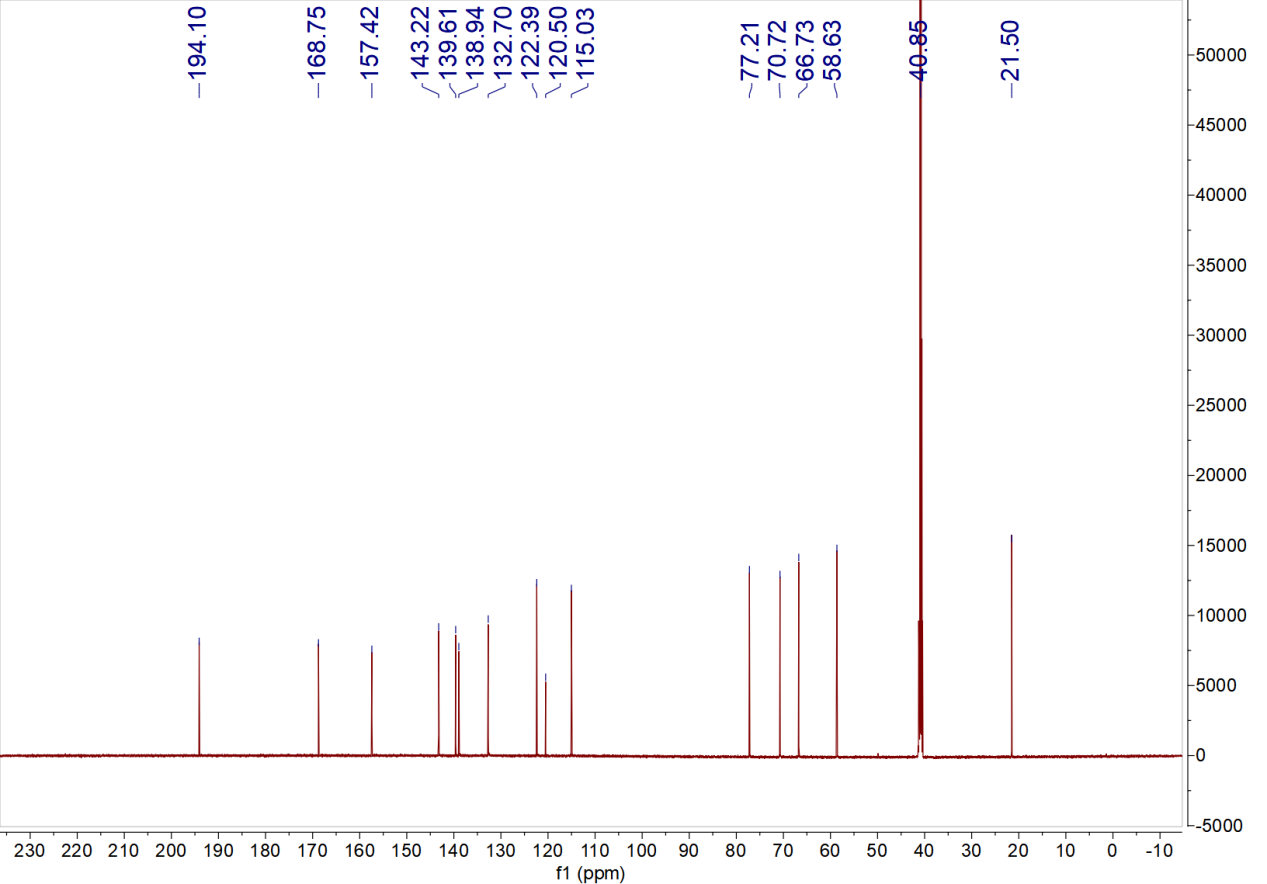


**Figure S8** The ^13^C NMR spectrum of **4** in DMSO-*d*_6_ (150 MHz).


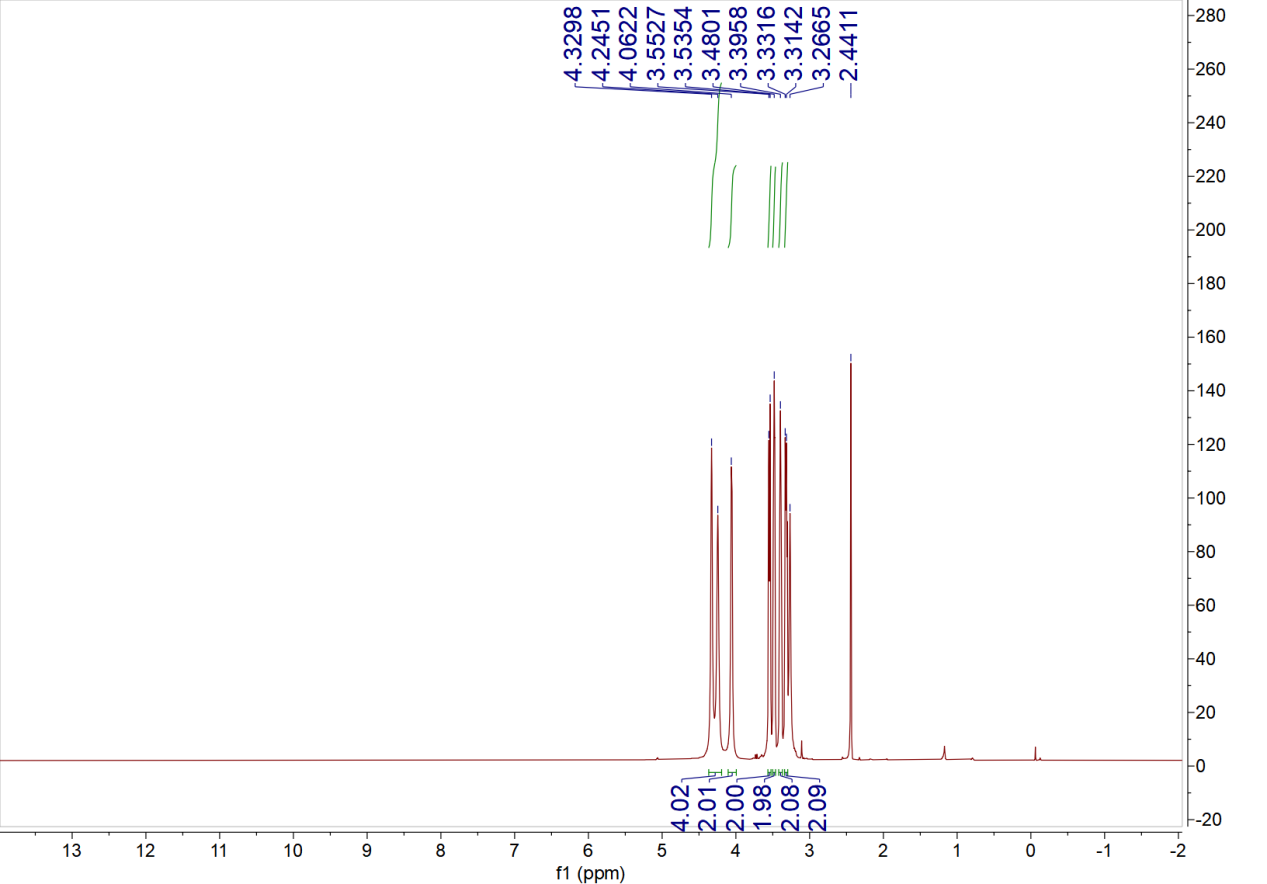


**Figure S9** The ^1^H NMR spectrum of **5** in DMSO-*d*_6_ (600 MHz).
